# Nature-Inspired Heat and Moisture Exchanger Filters Composed of Gelatin and Chitosan for the Design of Eco-Sustainable “Artificial Noses”

**DOI:** 10.1021/acsapm.3c00140

**Published:** 2023-04-12

**Authors:** Elisabetta Campodoni, Chiara Artusi, Brais Vazquez Iglesias, Alessia Nicosia, Franco Belosi, Alberta Vandini, Paolo Monticelli, Anna Tampieri, Monica Sandri

**Affiliations:** † Institute of Science, Technology and Sustainability for Ceramics (ISSMC-CNR), Faenza, RA 48018, Italy; ‡ Pollution S.r.l., Budrio, BO 40054, Italy; § Institute of Atmospheric Sciences and Climate (ISAC-CNR), Bologna, BO 40129, Italy; ∥ Institute of Microbiology, University of Ferrara, Ferrara 44121, Italy

**Keywords:** green chemistry, bioinspired material, HME device, circular economy, bacteriostatic

## Abstract

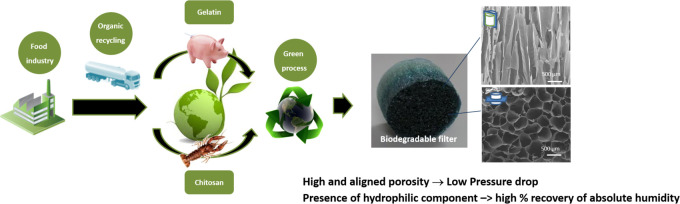

For long-term mechanical ventilation, during anesthesia or intensive care, it is crucial to preserve a minimum level of humidity to avoid damage to the respiratory epithelium. Heat and moisture exchange filters (HME), also called “artificial noses,” are passive systems that contribute to delivering inspired gases at about the same conditions of healthy respiration, i.e., 32 °C and relative humidity higher than 90%. Current HME devices suffer from limitations linked either to performance and filtration efficiency to their inadequate antibacterial efficiency, sterilization methods, and durability. Furthermore, in times of global warming and diminishing petroleum oil reserves, replacing the employing of synthetic materials with biomass biodegradable raw materials has considerable economic and environmental value. In the present study, a generation of eco-sustainable, bioinspired, and biodegradable HME devices are designed and developed through a green-chemistry process based on raw materials deriving from food waste and taking inspiration from the functioning, structure, and chemistry of our respiratory system. In particular, different blends are obtained by mixing aqueous solutions of gelatin and chitosan in various polymer ratios and concentrations and then by cross-linking them with different low amounts of genipin, a natural chemical cross-linker. Finally, the blends, post-gelation, are freeze-dried to obtain three-dimensional (3D) highly porous aerogels reproducing both the highly exposed surface area of the upper respiratory ways and the chemical composition of the mucus secretion covering the nasal mucosae. Results are comparable with accepted standards for HME devices and suitable bacteriostatic potential, thus validating these bioinspired materials as promising candidates to be used as an eco-sustainable generation of HME devices.

## 1 Introduction

In the 1950s, following the introduction of tracheostomy, the first prototype of the heat and moisture exchanger (HME) device was developed to make mechanical ventilation of patients during anesthesia and intensive care more accessible and more effective. When a patient is intubated, the trachea loses its warming, humidifying, and filtering functions of the upper airways as these are bypassed and replaced by an artificial medium. Therefore, the gas supplied to the patient must be artificially preconditioned to fill these lost functions without risking damage to the respiratory tract with a dry, cold gas. The initial idea was to deliver vaporized and heated air directly into the ventilator circuit connected to the patient. However, this led to numerous inconveniences, such as excessive condensation of water in the circuit, which often led to bacterial contamination and infections. In fact, several studies^1^ have reported a high incidence of nosocomial pneumonia caused by ventilation equipment contaminated with Gram-positive or negative bacilli. Furthermore, in addition to the infectious risk, the operating costs associated with this type of equipment were high.^2^ With this evidence, heat and moisture exchange devices (HMEs) have gradually become the only method of transporting and humidifying air in patients undergoing mechanical ventilation.^3^

For long-term mechanical ventilation, during anesthesia or intensive care, it is essential to maintain a minimum level of humidity to avoid damage to the respiratory epithelium and increased secretions. In this regard, the HMEs aim to maintain normal physiological conditions in the lower respiratory tract, conserving part of the heat and moisture of air exhaled by the patient to condition the inhaled gas by giving it the heat and humidity retained. HMEs are an effective, economical, and energy-efficient solution for the humidification of technical gases administered to patients.^4^ They can be compared to an “artificial nose,” with the ability to retain the stored humidity and the thermal energy of the exhaled gases, returning it, during inhalation, to the lower respiratory tract. HMEs can be divided into two categories: hygroscopic or hydrophobic. Hygroscopic HMEs feature a chemical-coated paper or fiber barrier that retains moisture and absorbs water on exhalation and releases it on inspiration, and they tend to saturate, which increases the inspiratory/expiratory resistance and reduces the heat and moisture retention efficiency.^5−7^ Instead, hydrophobic HMEs consist of a pleated hydrophobic membrane with small pores, which allows water to escape from the surface of the filter by surface tension without penetrating the holes below. Liquid water is unable to pass through them and only water vapor is retained from the gas. This second category of devices has lower resistance to flow and tends to be more efficient than hygroscopic HMEs.^8^ Furthermore, an air filter element can be added to the classic HMEs, which makes these modified devices filters as well as heat and humidity exchangers (HMEf). The removal of the liquid or solid particles (aerosols) from the gas takes place through electrostatic-based membranes (e.g., electrostatic filters) or using nanofibers (mechanical filters). Larger particles, failing to follow the path of the gas through the obstruction, collide with the filter by direct inertial impact or interception and lock onto the surface.^7^ Currently, the HMEs used in intensive care are based on technology from almost 20 years ago and therefore not very performing. Dellamonica et al. reported the comparison between 44 types of HMEs sold by different companies highlighting that the reasons why you should select one filter over another are not well defined and changed between anesthesia and intensive care. Further, the large availability makes it difficult to choose which test to use to assess filter efficiency and to compare them with each other.^9^ In general, they are made of synthetic materials, mostly polyurethane foams, or resistant materials, such as cellulose sheets.^4,6,9^

Nowadays, in a world where more and more efforts are being made to focus on green processes and environmentally sustainable materials, a major improvement in the performance of HMEs is needed to create more effective devices based on the use of natural sources. In particular, they should possess good hydrophilic and hygroscopic properties to accumulate the water vapor emitted by the patient during breathing and, at the same time, release moisture during the inhalation phase, ensuring a rapid exchange process.^10^ In addition, they should have excellent mechanical strength in both dry and wet conditions and be structured in such a way as to allow easy passage of air through the device and therefore comfortable breathing.^9^ Each property that the HME must possess to function properly requires an accurate selection of the constituting materials and the method to process them. The purpose of this work was to search for suitable natural low-cost sources whereby to develop innovative HME devices as a viable eco-sustainable alternative to those currently available on the market. To meet all of the requirements—rapid moisture and heat exchange, filtration ability but also structural stability, biodegradability, and low cost—a blend of natural polymers derived from waste recycling was sought and optimized.

The polymers were chosen following a biomimetic approach aimed at mimicking the chemical structure of glycoproteins, which represents one of the main components of mucus, a slippery secretion that covers our respiratory tract. It is composed of immunoglobulins, inorganic salts, protein, glycoprotein, and antiseptic enzymes. It is responsible for protecting the respiratory system by trapping allergens, pollutants, and bacteria and moisturizing and warming the inhaled air.

In particular, gelatin (Gel) extracted from pig skin, which mimics the main protein chain of glycoproteins, and chitosan (Chit) derived from crustaceans and mimicking their polysaccharide side chains were chosen for their chemistry and ideal characteristics to meet the needs of heat and humidity exchange and for the flexibility in the development of the three-dimensional (3D) porous structure. The chemical and structural stability of the device was instead controlled using a cross-linking reaction with genipin (Gen) extracted from the fruit of Gardenia jasminoides Ellis, which is able to interact with amino groups of Gel and Chit. This iridoid compound is well known and has been studied for a long time, e.g., as a treatment for diabetes mellitus or jaundice in traditional Chinese herbal medicine, and since it presents various functional groups in its structure, it has been used as a cross-linker to stabilize hydrogels in biomaterials development.^11^ For achieving a functional porous structure, the polymer blend composition was optimized and then freeze-dried to obtain aerogels with interconnected porosity by exploiting the vertical freezing and subsequent sublimation of water contained in the material.

In detail, Gel is a bio-based protein polymer coming from the hydrolysis of collagen, so completely biocompatible, nontoxic, biodegradable and, thanks to its plentiful alimentary use, has low cost and high availability. Gel is a promising material for HME development since it can create a 3D network showing good mechanical properties and high permeability to water vapor, optimal characteristics for the exchange of moisture.^12,13^ The cationic polysaccharide Chit instead, is extracted from the crustaceans, in particular, obtained after the deacetylation of chitin. It is mostly a waste material of the food industry, so it has, as well, low cost and high availability, in addition to being biocompatible and biodegradable. This natural polymer is particularly interesting in the HME development not only for its hydrophobicity but also for the ability to give easily a resistant porous structure, which is essential to allow the passage of air inside the filters. Moreover, it has an intrinsic antimicrobial activity, which is able to counteract the presence of bacteria, and in the formation of polymer blends, its cationic nature leads it to be an easily miscible polymer.^14−16^ In medium/high temperatures, Gel and Chit are soluble in water, so the passage of warm and humid air through the filter could cause the collapse of the structure. This point of weakness was bypassed by evaluating some chemical cross-linkers able to create irreversible covalent bonds, which lead to an increase of mechanical and chemical properties, and especially the resistance of the polymer in water.^17^ Many kinds of research demonstrated that the amino groups of collagen/gelatine and chitosan can react with genipin, forming intramolecular and intermolecular cross-linking networks.^18,19^ In this case, genipin was chosen as a cross-linker because of its ability to react with the amino functions of both Gel and Chit; creating a linkage between the two polymers improves the mechanical properties of HMEs.^20^ Finally, the freeze-drying process was crucial for obtaining an aerogel with an optimized porous structure, which allows for a high heat and moisture exchange and exerting a low drop in gas pressure and is suitable to function as an HME device.

## 2 Materials and Methods

### 2.1 Biopolymers

Gelatin (Gel) with mesh 4 and bloom 280 was extracted from pig skin from Italgelatine (Cuneo, Italy). Chitosan (Chit) of low molecular weight (75,774.77 g/mol)^21^ and deacetylation degree of 79 ± 1%^21^ was purchased by Sigma Aldrich (Saint Louis, Missouri). Genipin (Gen, purity 98%) of natural source was purchased by Wako Chemicals (Wako Pure Chemical) and obtained by extraction from Gardenia Jasminoides Ellis.

### 2.2 Synthesis of HME Filters

Biopolymer blends of different concentrations (1, 2, 3 wt %) have been obtained by mixing an aqueous solution of Gel and Chit to obtain Gel/Chit weight ratios of 70:30, 50:50, and 80:20, respectively.

**Figure 1:**
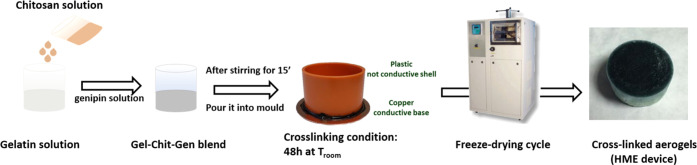
Synthesis and forming process steps to develop the Gel–Chit–Gen aerogel as an HME filter device.

In detail, the Gel solution was prepared by dissolving gelatin powder in water at 45 °C under magnetic stirring. The Chit solution was prepared by dissolving chitosan powder in acetic solution 0.1% (pH = 5.5) and stirring at room temperature till its complete dissolution. The Gen solution was prepared by dissolving genipin in water at room temperature under magnetic stirring. Gel was mixed with Chit, and then the Gen solution was added in different amounts to obtain a cross-link percentage of 0, 1, 2, and 4%. The whole blend was left to stir at room temperature for about 15 min obtaining its complete homogenization (Figure 1). The blend was poured into a 100 mL mold composed of a metal bottom and plastic walls (Ø = 5 cm) and closed from the bottom to avoid water evaporation during the 2 day cross-linking reaction.

The cross-linking reaction is confirmed by the color changing from yellow to blue after 48 h and the following transition from hydrogel to aerogel occurs through the freeze-drying cycle (*T*_freezing_ = −40 °C; first heating ramp = 5 °C/h up to −10 °C; second heating ramp = 1 °C/h up to 15 °C). After freeze-drying, the upper and the lower surfaces of the dried aerogel were cut off about 2 mm in thickness.^10^ Several parameters such as the blend compositions, polymer ratio, hydrogel concentration, cross-linker/polymer ratio, and freeze-drying process parameters, were tested and optimized to achieve an aerogel able to operate as an HME. All of the compositions are listed in the table below.

### 2.3 Characterization of HME Devices

Environmental scanning microscopy (ESEM, Quanta 200 FEG, FEI Company, Hillsboro, OR) was selected to analyze the morphology of the aerogels. Before starting the analysis, the samples tested need to be attached with carbon tape onto an Al stub and coated with a thin Au layer (Polaron Sputter Coater E5100 Equipment, Watford, Hertfordshire, U.K.).

The static contact angle was determined to evaluate the solid–liquid interfacial tension of the materials and the analyses was performed using a tensiometer (Video-Based Optical Contact Angle Meter OCA 15+, Innovent, Filderstadt, Germany) as described previously.^22^ Values were expressed as the mean ± standard error (*n* = 10).

The weight loss test allows us to evaluate the stability of the scaffold by measuring the degradation percentage. The test was performed by continuously shaking the samples in aqueous conditions at 37 °C; at specific time points, samples were washed twice, freeze-dried, and weighed. Finally, eq 1 was used to evaluate the cross-linking percentage.^12,23^
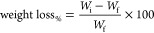
1where *W*_i_ is the initial weight of the dried sample and *W*_f_ is the weight of the freeze-dried sample at specific time points.

The cross-linking degree due to genipin was evaluated by the 2,4,6-trinitrobenzenesulfonic acid (TNBS) assay, using a UV–vis spectrophotometer (Perkin-Elmer Lambda 35, Milano, Italy) as described previously.^13^

#### 2.3.1 Moisture Recovery

For the moisture recovery tests, the test apparatus used was based on the lung model described in ISO 9360 and consisted of two separate circuits (Figure S1) as described previously, and the percentage recovery of absolute humidity (AHrec) was calculated with eqs 2 and 3.^24^
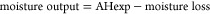
2
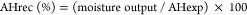
3where moisture loss is the weight difference of the test apparatus over a certain period and AHexp is the absolute humidity of expired air, assuming flow was fully saturated with water vapor.

#### 2.3.2 Pressure Drop

The test apparatus used in the moisture recovery test allows us to evaluate the pressure drop of the samples before and after 2 h of preconditioning. 30, 60, and 90 L/min of dry air were selected according to ISO standard 9360. Resistance across the sample holder was recorded by an electronic differential manometer (2080P, Digitron, United Kingdom).

#### 2.3.3 Bacteriostatic Activity

A quantitative surface test carried out according to the EN 13697:2015 standard method procedure was performed to evaluate if the sample can oppose itself to the proliferation of bacteria [EN 13697-2015. Chemical disinfectants and antiseptics—quantitative nonporous surface test for the evaluation of the bactericidal and/or fungicidal activity of chemical disinfectants used in food, industrial, domestic, and institutional areas—test method and requirements without mechanical action (phase 2, step 2)].^10^

The in vitro test was conducted using American Type Culture Collection (ATCC) strains of bacteria, such as *Escherichia coli* ATCC 8739, *Pseudomonas aeruginosa* ATCC 9027, *Staphylococcus aureus* ATCC 6538, *Staphylococcus epidermidis* ATCC 27626, and fungi, such as *Candida albicans* ATCC 10231 (yeast) and *Aspergillus brasiliensis* ATCC 16404 (mold), as microorganisms tested.

Each test was carried out at 2020 ± 1 °C and the contact time chosen was 1, 4, 24, and 72 h. The test was performed separately by contaminating each surface (upper and inner) of the samples with 1 mL of each microbial suspension. The microbial title chosen for the bacteria suspension is 106 CFU/mL, while that for the fungi suspension is 105 CFU/mL. Each sample was contaminated in the presence of a specific culture media; furthermore, the control was prepared by contaminating empty plates. After 72 h at 37 °C, samples were neutralized and the difference between the number of organisms subtracted from the samples with respect to those from the controls was calculated as a log10 reduction to evaluate the bactericidal properties of the samples tested.^10^

## 3 Results and Discussion

Taking inspiration from mucus chemical composition and, in particular, from the glycoprotein structure, which are proteins containing oligosaccharide chains covalently attached to the amino acid side chains, Gel and Chit biopolymers were chosen for the development of HME devices since they are low-cost and they have a moisturizing function very close to natural mucus.^17,25−27^ Gel is a protein extracted from the waste of various animals that features a good water affinity and the ability to confer elasticity and hydrophilicity once added into a blend.^10,28,29^ Chit is a natural polysaccharide derived from the deacetylation of chitin, which is able to add reinforcement and strength to the blend and confer fairly good antibacterial properties.

Different amounts of Gen, selected as a biocompatible and nature-derived chemical cross-linker, were considered to modulate the chemical stability in dry and wet environments and increase the Gel–Chit interconnection.^10,30^ As already reported in the literature, during the cross-linking treatment with Gen, gelatin and chitosan can react with their amino groups and cross-link with each other to form an interpenetrating polymer network (IPN) that is revealed through color changing from clear to blue, meaning that a spontaneous reaction between polymers occurred (Figure S2).^10,25,31,32^

Samples with different Gel/Chit _wt_% ratios and various hydrogel concentrations were developed and the effect of their morphological structure, degradation, and performances was evaluated.

**Figure 2:**
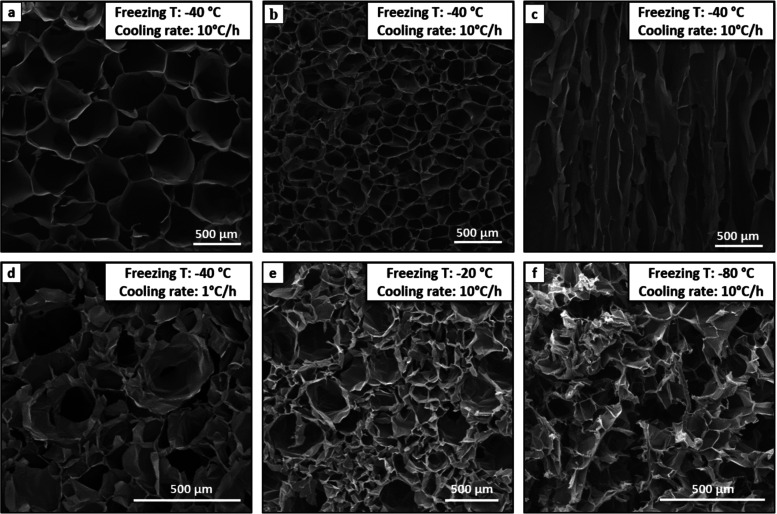
SEM morphologies of sample G: hydrogel concentration 2 _wt_%, Gel/Chit _wt_% ratio 70:30, Gen 2 _wt_%: (a) upper transversal section; (b) lower transversal section; (c) sagittal section of sample G (*T*_freezing_ = −40 °C; cooling rate = 10 °C/h); (d) transversal section of sample G (*T*_freezing_ = −40 °C; cooling rate = 1 °C/h); (e) transversal section of sample G (*T*_freezing_ = −20 °C; cooling rate = 10 °C/h); and (f) transversal section of sample G (*T*_freezing_ = −80 °C; cooling rate = 10 °C/h).

**Figure 3:**
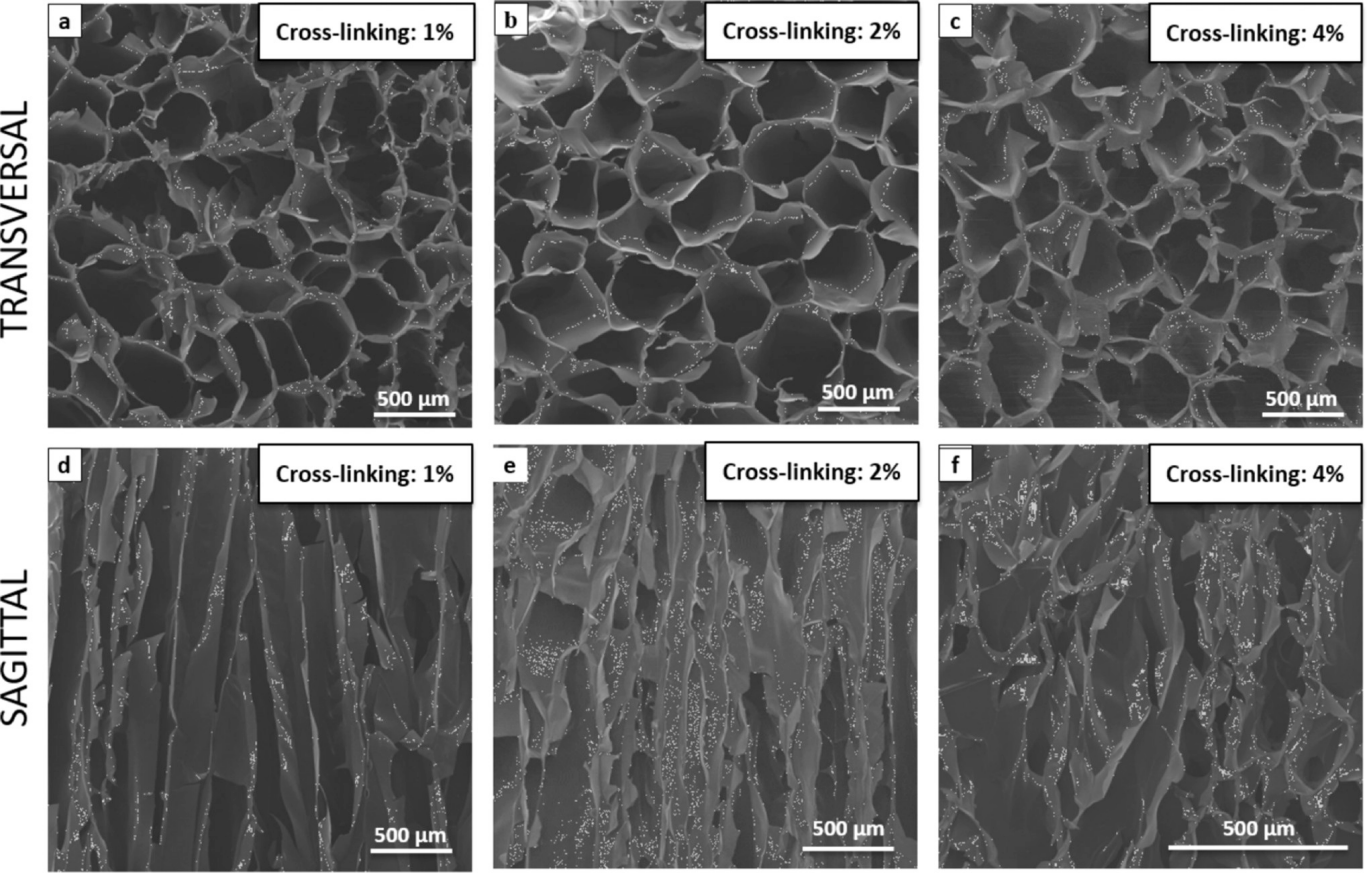
SEM morphologies of sample A, hydrogel concentration 2 _wt_%, Gel/Chit _wt_% ratio 70:30, Gen 1 _wt_%: (a) transversal section and (d) sagittal section; sample G: (b) transversal section and (e) sagittal section; and sample B, hydrogel concentration 2 _wt_%, Gel/Chit _wt_% ratio 70:30, Gen 4 _wt_%: (c) transversal section and (f) sagittal section.

**Figure 4:**
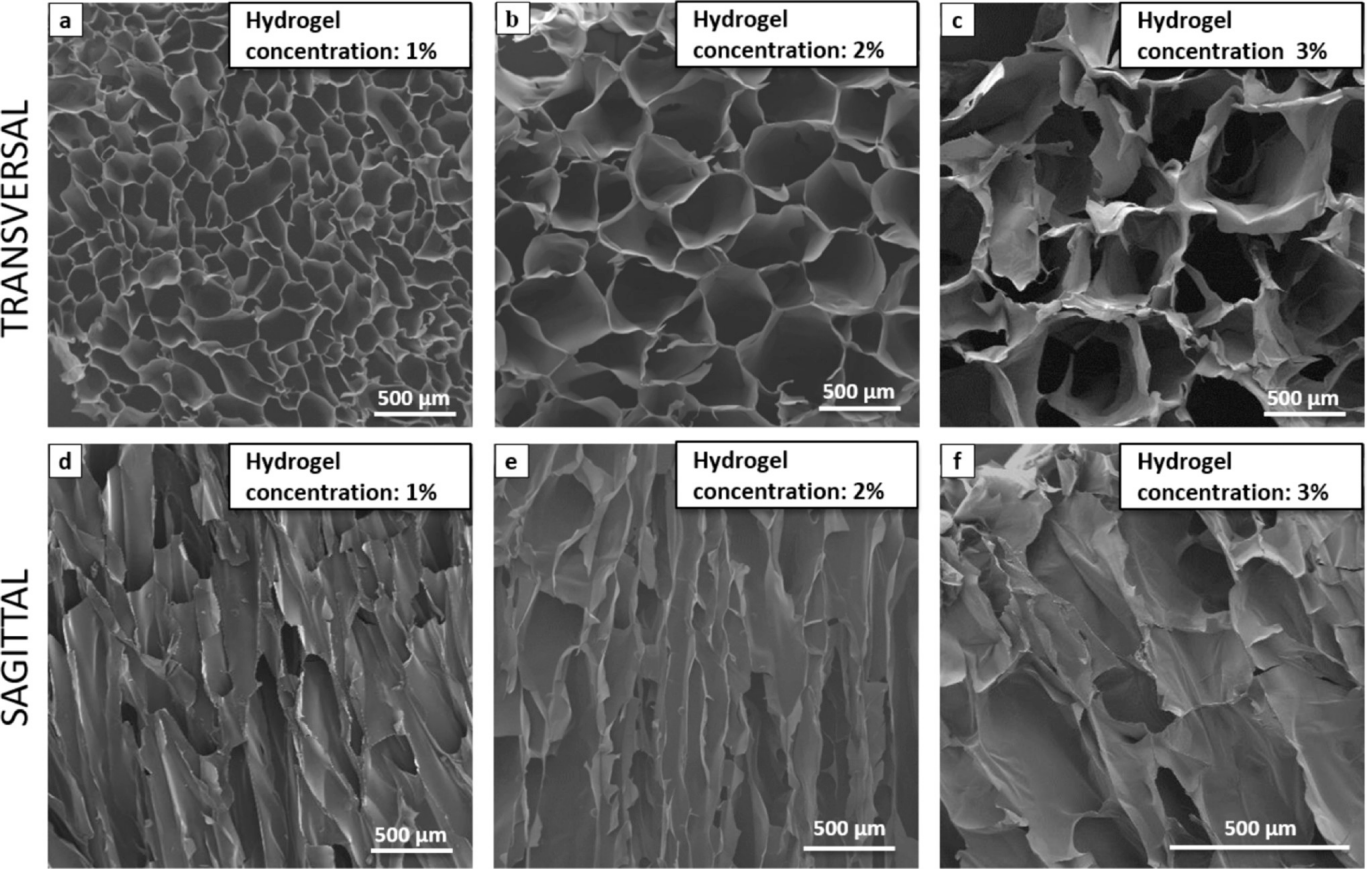
SEM morphologies of sample C, hydrogel concentration 1 _wt_%, Gel/Chit _wt_% ratio 70:30, Gen 2 _wt_%: (a) transversal section and (d) sagittal section; sample G, hydrogel concentration 2 _wt_%, Gel/Chit _wt_% ratio 70:30, Gen 2 _wt_%: (b) transversal section and (e) sagittal section; and sample D, hydrogel concentration 3 _wt_%, Gel/Chit _wt_% ratio 70:30, Gen 2 _wt_%: (c) transversal section and (f) sagittal section.

**Figure 5:**
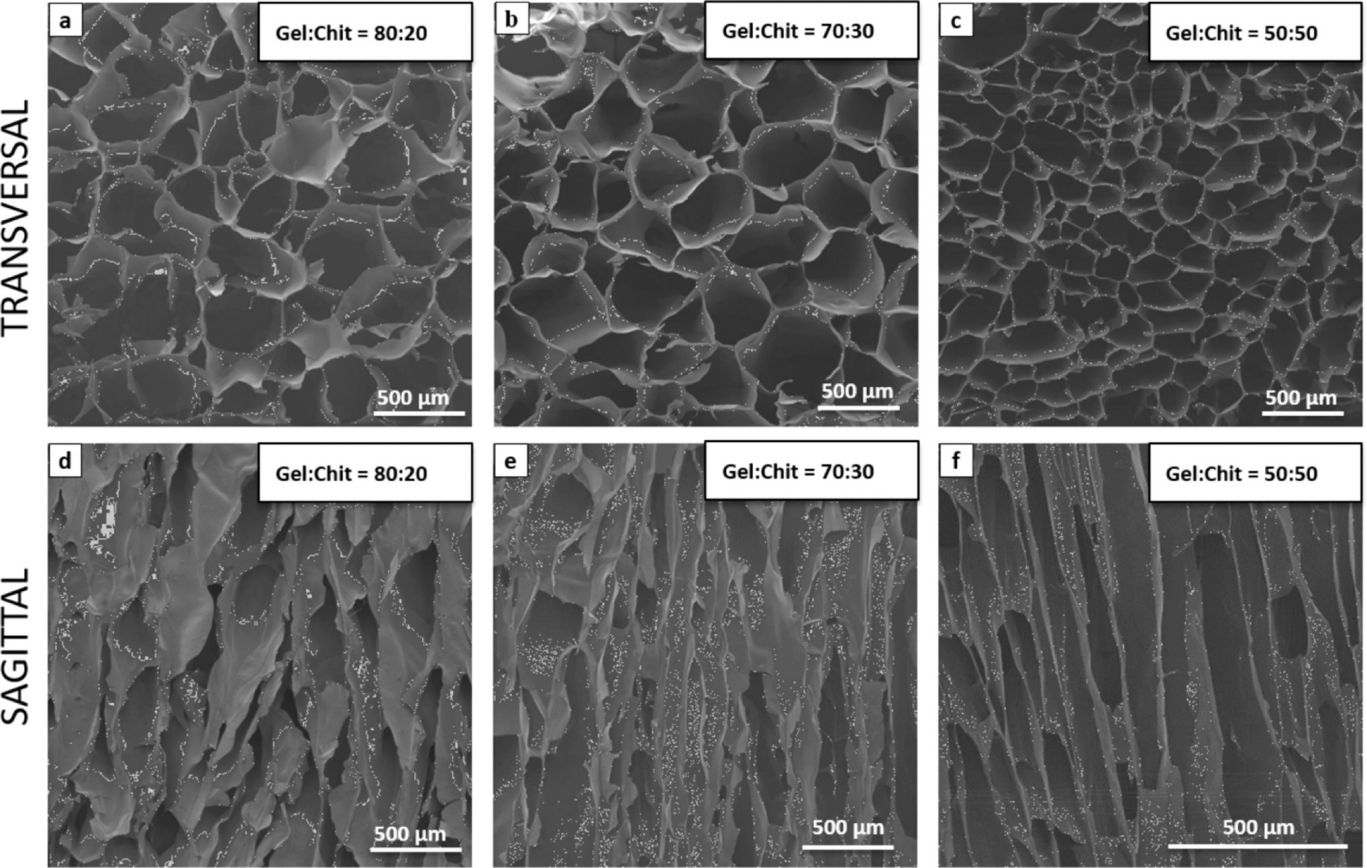
SEM morphologies of sample E, hydrogel concentration 2 _wt_%, Gel/Chit _wt_% ratio 80:20, Gen 2 _wt_%. (a) transversal section and (d) sagittal section; sample G, hydrogel concentration 2 _wt_%, Gel/Chit _wt_% ratio 70:30, Gen 2 _wt_%. (b) transversal section and (e) sagittal section; and sample F, hydrogel concentration 2 _wt_%, Gel/Chit _wt_% ratio 50:50, Gen 2 _wt_%: (c) transversal section and (f) sagittal section.

From scanning electron microscopy (SEM) images (Figures 2–5), it was possible to study those variables that affect the aerogel morphology. In this work, four different variables were considered and optimized to achieve the required structure and behavior of the device: freezing temperature and cooling rate, cross-linking degree, hydrogel concentration, and polymer _wt_% ratio (Gel/Chit).

First, pore size and orientation were significantly affected by freezing temperature and cooling rates. Figure 2 (panel a–c) shows the upper and lower transversal sections and sagittal section of sample G (Table 1) freeze-dried, applying a cooling rate of 10 °C/h and a freezing temperature of −40 °C. The pore size is homogenous but increases from the bottom (Figure 2b) to the top (Figure 2a) of the sample, and it is due to the vertical freezing typical of the freeze-drying technique. The sagittal section revealed elongated pores vertically oriented (Figure 2c) along the whole sample. However, cutting off both the sample’s surfaces is possible to obtain homogenous devices with an anisotropic interconnected porosity featured by vertically aligned channels and uniform pore size, an essential condition for the creation of an HME device with reduced dead space and pressure drop.

**Table 1:** Composition and Code of the Developed Gel/Chit-Based HMEs

sample	hydrogel concentration [_wt_%]	gel/chit _wt_ ratio	amount of gen [_wt_%]
NCL	2	70:30	0
A	2	70:30	1
B	2	70:30	4
C	1	70:30	2
D	3	70:30	2
E	2	80:20	2
F	2	50:50	2
G	2	70:30	2

Instead, as shown in Figure 2, panels D–F, a too low cooling rate (1 °C/h; −40 °C) (Figure 2d) and a relatively high freezing temperature (10 °C/h; −20 °C) (Figure 2e) lead to obtaining porous structures endowed with no homogeneous pore diameter and no effective preferential orientation. However, a too low freezing temperature (10 °C/h; −80 °C) (Figure 2f) generates evenly small pores, but they are randomly oriented.

It is well known that, during freeze-drying, the rate of nucleation of ice crystals and the rate of heat diffusion affect the distribution of the dissolved polymer, defining the amount of ice crystals formed and their size and orientation, respectively.^10,33,34^ Therefore, with a too low freezing temperature (−80 °C), a large number of small ice crystals are formed that will grow little, leading to an aerogel with a smaller pore size after freeze-drying.^34−36^ In addition, the direction and the speed of heat transfer influence the shape and orientation of ice crystals since the rapid cooling by direct contact with the freezing plate will orient the pores due to a temperature gradient included in the chitosan–gelatin hydrogel, leading to the formation of columnar ice crystals (Figure 2f).^37^ On the other hand, a lower cooling rate and/or a too high freezing temperature decrease the vertical temperature gradient resulting in a horizontal growth of some ice crystals nuclei (Figure 2d,f).^10,35^

Analyzing the effect of the cross-linking degree (Figure 3a–f, in increasing order of reticulation), it is possible to observe that no significant changes in the pore size are observed, but they are evident in their morphology. As shown in Figure 3a–d, sample A highlights some network breaks in the transversal section and the pore walls are thinner and more breakable (Figure 3d). On the other hand, sample B shows pore walls much thicker (Figure 3f) because an increase in the linkage between the polymer chains leads to a more resistant material. In addition, the monodirectionality of the channels decreases with the increase of the cross-linking degree. This effect could be explained by the increased viscosity of the more cross-linked hydrogel, which is unfavorable to the movement of water and the molecular chains within the gel during freezing.^10^ Therefore, the ice crystals grow with more difficulty inside the highly viscous gel than in those less viscously cross-linked.^35,38^

The concentration of the Gel/Chit hydrogel is another important parameter with a strong effect on the average pore size, the thickness of their wall, and consequently, also on the vapor uptake capacity of the aerogel. With the same volume of the freeze-dried Gel/Chit solution, a lower concentration of Gel (Figure 4a,c) corresponds to thinner pore walls and smaller pore sizes. This occurs because the higher concentration of the hydrogel hampers the growth of ice crystals and allows the formation of few ice nuclei rather than those formed in the gel with a lower concentration, in which Gel and Chit molecules are subjected to reduced resistance and the ice crystals can grow more quickly and straighter.^35,39,40^

As reported in previous research, the higher viscosity of the solution led to unfavorable moving into the solution of both water and molecular chains.^41^

Other significant changes occurred concerning pore size and morphology by changing the polymer (Gel/Chit) ratio. A greater amount of Gel leads to an aerogel with a more disordered morphology (Figure 5a,d), while a greater percentage of Chit leads to a more tailored structure (Figure 5f); this is because Gel is a macromolecule and tends to form cross-linkage with chitosan molecules through hydrogen bonding and electrostatic interactions. For this reason, if the Gel/Chit ratio is lower, aerogel tends to freeze-dry, arranging in packaged and more ordered layers, while if the Gel/Chit ratio is higher, scaffolds tend to arrange with more globular and randomly arranged pores.^41^

**Figure 6:**
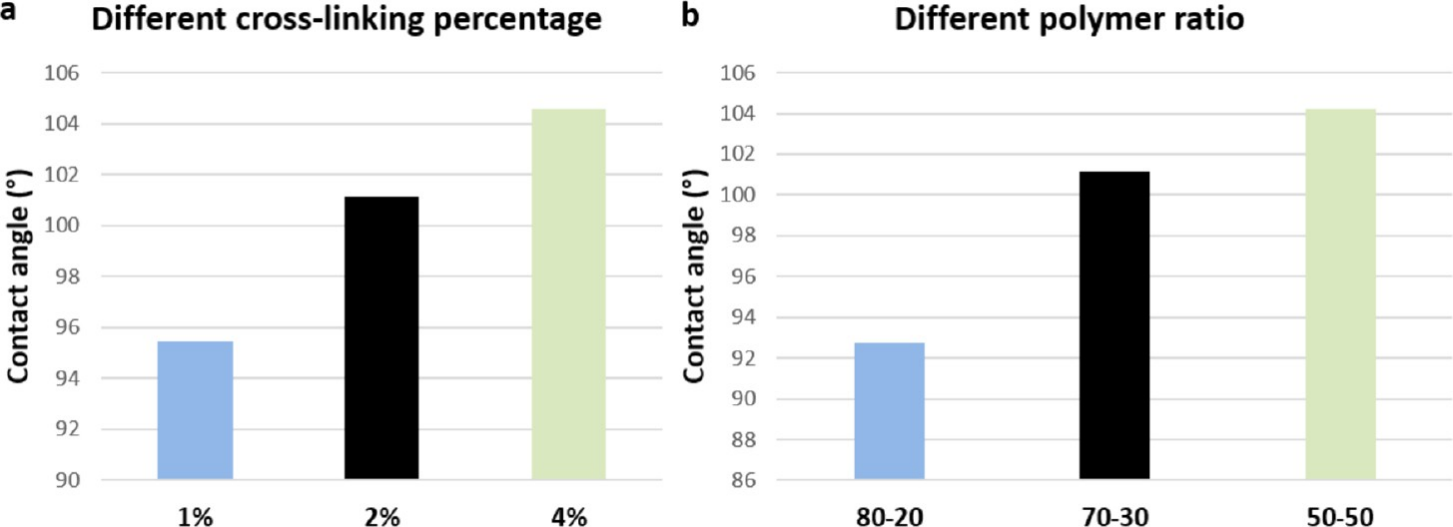
Contact angles of samples with (a) different cross-linking percentages (from 1 to 4%) and (b) different polymer ratios (Gel/Chit from 80:20 to 50:50).

The wettability of the various compositions of Gel/Chit was measured using a static water contact angle on dried films and the results are reported in the graphs in Figure 6.

All samples showed medium contact angles in the range from 90 to 105°, highlighting poor hydrophilicity (Figure 6). By observing the trend of the contact angle value, it is clear that the cross-linking degree and the _wt_% content of Chit allow controlling these values. The less cross-linked blend shows greater hydrophilicity (sample A) and with the increase of the percentage of genipin (samples G and B), which determines the formation of a more closed molecular network through the formation of covalent bonds involving free amino groups, the water contact angle increases, which means a decrease in water affinity.^13,20,42^

It is also clearly observable that higher amounts of Chit lead to an increase in the contact angle and therefore to a decrease in the hydrophilicity of the samples. This finding is attributed to the greater hydrophilicity of Gel compared to that of Chit,^43^ and this determines that an increase in the more hydrophilic component (Gel) corresponds to an overall increase in the hydrophilicity of the sample (Figure 6b).

Since the analysis was carried out on dried films, samples C and D, differing from sample G only in the polymer concentrations, were not tested because the chemical composition was unchanged.

The TNBS method^13^ has been used to evaluate the effective cross-linking degree of the Gel/Chit blends with genipin recording the amount of free primary amine group of the samples in comparison with the not cross-linked sample. In detail, only those samples with different cross-linking percentages were evaluated (samples A, B, and G) and the obtained results are reported in Table 2.

**Table 2:** TNBS Test to Evaluate the Cross-Linking Degree of Samples A, G, and B (from 1 to 4%)

sample	cross-linker percentage (%) (genipin/polymer)*100	cross-linking degree (%)
A	1	56
G	2	78
B	4	81

It is clear that the genipin/polymer ratio of 1 _wt_% resulted in the lower cross-linking degree and could not be enough for the achievement of an efficient reticulation; between 2 and 4 _wt_%, instead, no big differences in the degree of cross-linking were highlighted. These results lead to identifying 2 _wt_% as the amount needed to obtain a stable aerogel. As the data have suggested, the increase in the genipin amount up to 4 wt % did not significantly affect the effective cross-linking reaction.

Degradation tests were conducted in Milli-Q water at pH 7 and 4 and at 37 °C to better simulate the physiological condition of the breath.^12^ Although the HME filters have a usage time of up to one week, the test was conducted up to three weeks on samples cross-linked with 1, 2, and 4 _wt_% of the genipin/polymer ratio (samples A, G, and B), samples obtained from a hydrogel concentration of 1, 2, and 3 _wt_% (samples C, G, and D), and samples with a Gel/Chit _wt_ ratio of 80:20, 70:30 and 50:50 (samples E, G, and F).

**Figure 7:**
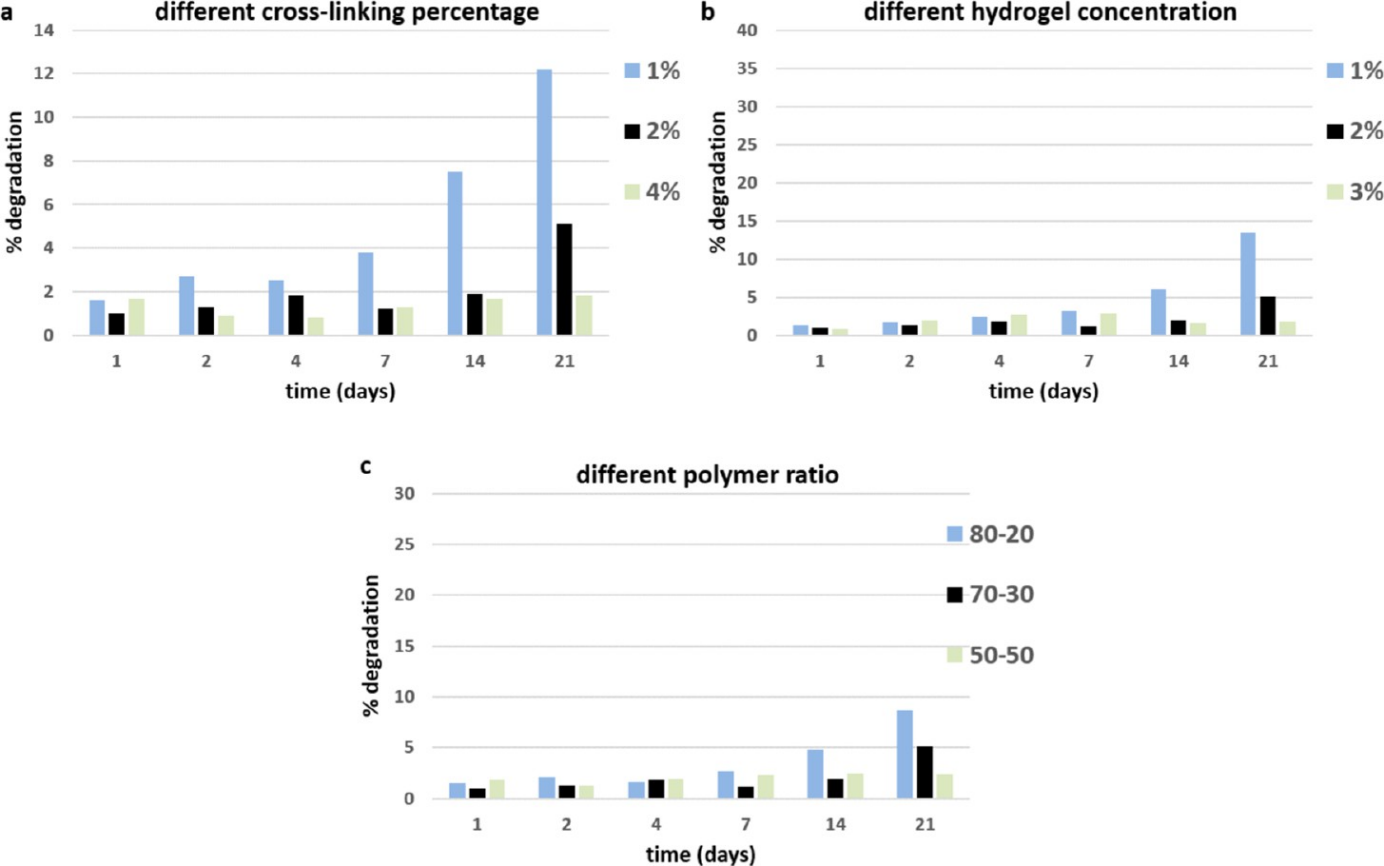
Wt % degradation test of samples with different (a) cross-linking percentages from 1 to 4 wt % (samples A, G, and B); (b) hydrogel concentrations from 1 to 3 wt % (samples C, G, and D); and (c) Gel/Chit polymer wt ratios from 80:20 to 50:50 (samples E, G, and F).

Although all of the samples degraded less than 5% in the first week, in the following two weeks, the degradation profile significantly changed (Figure 7). Samples A, C, and E show greater degradation than the other samples. What is observed is that the stability of aerogels in aqueous media increases proportionally to the amount of genipin, highlighting the chemical stabilization of the aerogel due to the cross-linking process.

The increase of the aerogel stability when 4 wt % of genipin is introduced in comparison with 2 wt % of genipin highlights the complete reaction without any unreacted cross-linker in the samples where 2 wt % of genipin was chosen. Another important parameter influencing the aerogel degradation in the water medium is the hydrogel concentration because a lower hydrogel concentration results in a more porous aerogel and thus faster degradation. In fact, after two weeks, the aerogel obtained from the less concentrated hydrogels and cross-linked with 2 _wt_% of genipin (sample C) starts to dissolve faster, a sign that a higher porosity affects the aerogel stability and, on the other hand, that the cross-linking extends the stability of the material without compromising its biodegradability. Finally, chitosan promotes the stability of the samples due to its natural insolubility at a pH above 6.5 (Figure 7c).

In conclusion, all of the previously reported analyses confirmed that through the optimization of the various synthesis parameters, it is possible to modulate the aerogel properties and prepare samples displaying the required hydrophilicity and pores with ideal dimensions, morphology, and interconnection essential to promote an efficient moisture exchange without induce a high pressure drop. Furthermore, thanks to the chemical cross-linking with genipin, the devices remain stable over time, allowing their storage and prolonged use.

Based on this, some samples’ compositions were evaluated in terms of moisture exchange efficiency and pressure drop. This test was crucial for the selection of the most suitable material because, following the ISO 9360 standards, the minimum accepted values of the moisture output are above 30 mgH_2_O/L (80 _wt_% of recovery of absolute humidity) and for the pressure drop, the higher accepted value is 5 mbar.^6,9^

**Figure 8:**
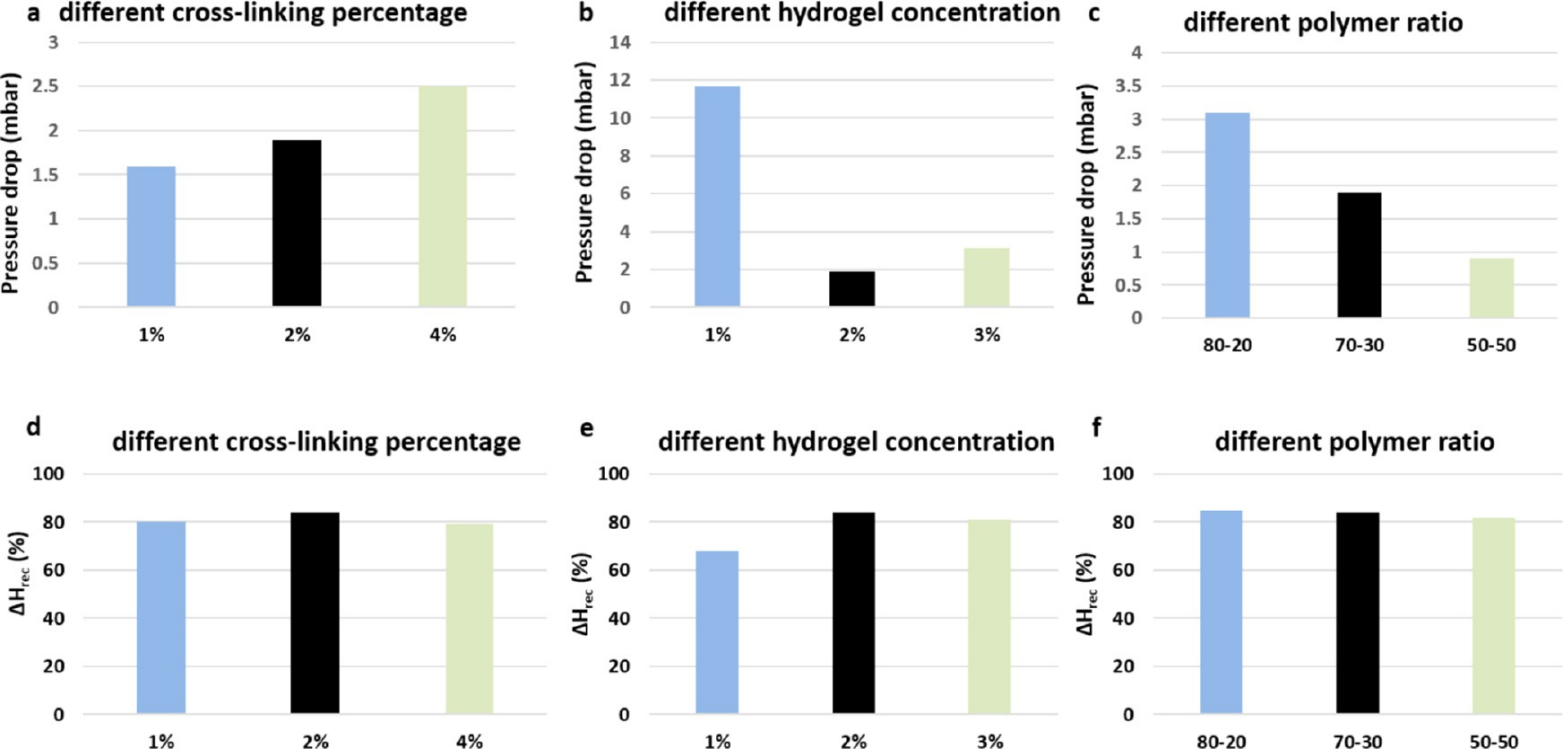
Comparison from the pressure drop (a–c) induced by samples and moisture exchange of samples (d–f) with different (a–d) cross-linking percentages from 1 to 4 _wt_% (samples A, G, and B); (b–e) hydrogel concentrations from 1 to 3 _wt_% (samples C, G and D); and (c, d) polymer wt ratios from 80:20 to 50:50 (samples E, G, and F).

The values of the pressure drop of the various samples are shown in Figure 8. No large variations were observed by changing the genipin amounts from 1.6 to 2.5 mbar; this finding can be attributed to similar pore morphology and size in the samples (Figure 8a). Also, SEM images show that all samples with different cross-linking percentages (samples A, G, and B) are characterized by pores with a channel-like morphology, even if B showed a less ordered structure in comparison with A and G.

From the data acquired on samples with different polymer ratios (Figure 8b) emerged a clear effect of the porous structure on the pressure drop: the most ordered and aligned structure highlighted from sample F (Figure 5) determines a lower pressure drop than the two other samples E and G, although the difference is not huge. Finally, comparing the values of pressure drop registered in samples obtained from hydrogels with different concentrations instead, we noted some evident variations (Figure 8c) due to the sample’s pore size and morphology. As a matter of fact, all samples showed a porosity range between 94 and 97% highlighting as all samples are highly porous and their porosity does not influence the pressure drop or other performance of the HME. Conversely, although all samples show high porosity and pore size and, in particular, their morphology and distribution significantly influence the pressure drop and other performances of the HME, sample C, indeed, showed very high resistance to airflow; it registered a pressure drop higher with respect to sample G (11.5 and 1.9 mbar, respectively). This phenomenon could be explained by the very closed and irregular structure of this sample observed by SEM images (Figure 4) which can obstruct the air passage through the filter. It was possible to observe smaller pores in sample C with respect to sample G, and channel mergers often interrupt the vertical path of the little pores.

The moisture exchange efficiencies (ΔHrec) show only little changes by varying the different parameters (Figure 8). In the function of the cross-linking process, a medium value of the cross-linker (2 _wt_% of sample G) results to be the optimal condition for balancing the hydrophobic/hydrophilic properties in the aerogel, so the percentages of moisture exchange are 84% in sample G, 80% in sample A, and 79% in sample B (Figure 8d). By changing the hydrogel concentration (Figure 8e), the lower efficiency is registered for sample C, while no significant differences were registered between samples G and D. This result can be explained by taking into account that the moisture exchange is affected by the chemistry of the material and also by the pressure drop induced by the morphology of the aerogel; therefore, if the air outlet is smaller, the overall moisture exchange will also be less. Finally, regarding the polymer ratio (Figure 8f), chitosan adversely influences the moisture exchange capacity of the device, though with no significant differences.

In general, the achieved results of water vapor retention are in line with the accepted standards for HME devices, demonstrating that these kinds of bioinspired aerogels are promising to be used as the next generation of HME devices. By the end, comparing the features highlighted in the previous tests, sample G has been selected as the most promising material to be inserted as a filtering component in an HME device.

The evaluation of the bactericide/bacteriostatic properties is fundamental and required for the HMEs used in hospitals because no proliferation of bacteria over or inside HME is allowed.

Moreover, a preliminary evaluation of the bacteriostatic potential was carried out; in fact, in filtering devices for hospital uses, bactericide/bacteriostatic properties are very significant and required to avoid the proliferation of bacteria over and inside the filter.^10^ According to the EN 13697:2015 standard^a^, the test was conducted by contaminating the surface and the internal samples’ section with different microbes; in Table 3, the percentage of growing inhibition is reported.

**Table 3:** Inhibition of Microbial Growth for the Surface and Internal Section of Sample G

	superficial section				internal section			
	contact time				contact time			
microbes	1 h	4 h	24 h	72 h	1 h	4 h	24 h	72 h
*Escherichia coli*	7%	7%	14%	14%	29%	29%	30%	31%
*Pseudomonas aeruginosa*	0%	0%	0%	17%	0%	17%	17%	17%
*Staphylococcus aureus*	8%	54%	95%	>99%	23%	75%	95%	>99%
*Staphylococcus epidermidis*	14%	80%	95%	>99%	14%	79%	96%	>99%
*Candida albicans*	17%	17%	50%	93%	17%	25%	67%	97%
*Aspergillus brasiliensis*	0%	0%	17%	17%	0%	0%	17%	17%

The specific test was conducted on the surface and in an internal section of the filters, and the percentage of growing inhibition of several tested microbes is reported in Table 3.

It evaluated bacteriostatic activity against Gram-negative bacteria, coliforms, and also against opportunistic pathogen yeast such as *E. coli* ATCC 8739, *P. aeruginosa* ATCC 9027, *S. aureus* ATCC 6538, *S. epidermidis* ATCC 27626, *C. albicans* ATCC 10231, and *A. brasiliensis* ATCC 16404.

These microorganisms represent the main nosocomial pathogenic indicators: *E. coli* Gram-negative bacteria, belonging to the group of enterobacteria and is the main indicator of fecal contamination (defined as coliform); *P. aeruginosa* Gram-negative bacteria is primarily an opportunistic nosocomial pathogen and it, therefore, produces infections especially in hospitalized patients, preferring those who are debilitated, immunocompromised, or undergoing mechanical ventilation; *S. aureus* and *S. epidermidis* Gram-positive bacteria, belonging to the genus of staphylococci typically present in the skin and mucosa; *C. albicans* saprophytic yeast, which normally resides in the oral cavity, intestines, and vagina and causes endogenous infections; and *A. brasiliensis* ubiquitous, airborne mold, which causes severe lung infections.^10^

In vitro quantitative tests by direct contact highlighted the efficient inhibition of different microbial growth in sample G, thus, demonstrating its bactericidal or bacteriostatic activity against the majority of microorganisms in time.

Sample G showed, in addition, a fungistatic effect against molds belonging to the *Aspergillus* genus, while it displayed very good bactericidal activity against Gram-positive bacteria, like *Staphylococcus*, with a reduction of 95% after 24 h of contact.

## 4 Conclusions

An eco-sustainable filtering component suitable for the production of innovative HME devices was successfully designed and developed. For this achievement, gelatin, chitosan, and genipin, as a cross-linking agent, were selected to prepare a homogeneous blend in order to create, after freeze-drying, a 3D porous aerogel with suitable chemical properties and an organized porous structure. The hydrogel concentration and the freeze-drying process parameters have been chosen with the aim of getting a structure with a low-pressure drop, allowing comfortable breathing and respecting the requirement of the reference standard. The polymer ratio and cross-linking percentage were selected to obtain a stabilized structure and a higher moisture exchange efficacy. Consequently, the filters developed here are characterized by optimized moisture exchange, low pressure drop, affordable cost, antimicrobial efficiency, and biodegradability and can be sterilized by γ-rays. Furthermore, because the filter composition and structure do not vary with temperature, UV light, and humidity, their shelf-life is remarkably longer, and their packaging is simplified. All of these advantageous properties lead to the reduction of handling costs and waste treatment and offer a significant industrial and economic opportunity.

## Supporting Information Available

Schematic diagram of the experimental test apparatus based on ISO 9360 (Figure S1) and cross-linking reaction between Gel and Chit activated from Gen molecules through a reaction with their amino groups (Figure S2) (PDF)

## Footnotes

a EN 13697-2015. Chemical disinfectants and antiseptics—quantitative nonporous surface test for the evaluation of bactericidal and/or fungicidal activity of chemical disinfectants used in food, industrial, domestic, and institutional areas—test method and requirements without mechanical action (phase 2, step 2)-*Online+. Available: https://www.en-standard.eu.
