# Supplementary material for: Nature-Inspired Heat and Moisture Exchanger Filters Composed of Gelatin and Chitosan for the Design of Eco-Sustainable “Artificial Noses”
Source: ACS Appl Polym Mater. 2023 Apr 12;5(5):3468–79. doi: 10.1021/acsapm.3c00140 (PMC10186330; doi:10.1021/acsapm.3c00140)
Supplement: Supplementary file 1 — ap3c00140_si_001.pdf [file ap3c00140_si_001.pdf]

Nature-inspired heat and moisture exchange filters composed of gelatin and chitosan for the design of eco-sustainable artificial noses

Elisabetta Campodoni[a], Chiara Artusi[a], Brais Vazquez Iglesias[b], Alessia Nicosia[c], Franco Belosi[c], Alberta Vandini[d], Paolo Monticelli[b], Anna Tampieri[a], Monica Sandri[a*]*

[a] Institute of Science, Technology and Sustainability for Ceramics (ISSMC-CNR), Faenza (RA), 48018, Italy

[b] Pollution S.r.l., Budrio (BO), 40054, Italy

[c] Institute of Atmospheric Sciences and Climate (ISAC-CNR), Bologna (BO), 40129, Italy

[d] Institute of Microbiology, University of Ferrara, Ferrara, 44121, Italy

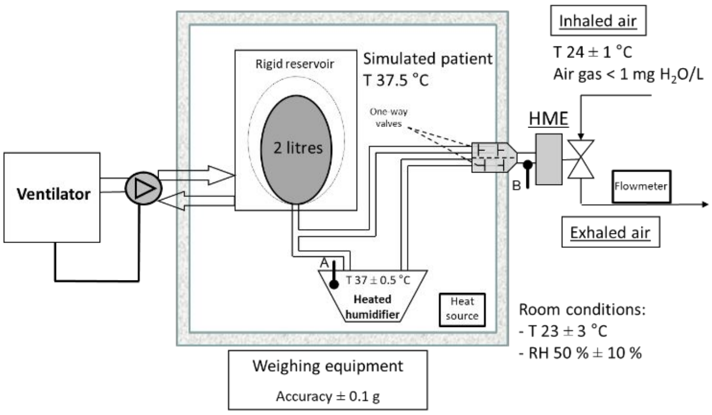

Figure S1. Schematic diagram of the experimental test apparatus based on ISO 9360

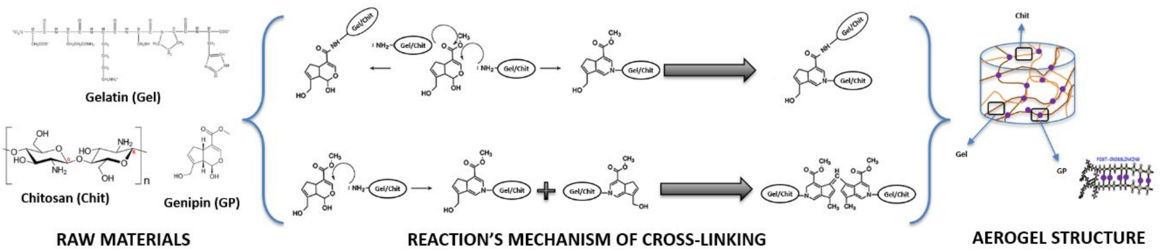

Figure S2 Cross-linking reaction between Gel and Chit activated from Gen molecules thought reaction with their amino groups.
